# Diagnosis of Occlusal Tooth Wear Using 3D Imaging of Optical Coherence Tomography Ex Vivo

**DOI:** 10.3390/s20216016

**Published:** 2020-10-23

**Authors:** Misa Kashiwa, Yasushi Shimada, Alireza Sadr, Masahiro Yoshiyama, Yasunori Sumi, Junji Tagami

**Affiliations:** 1 Department of Cariology and Operative Dentistry Department, Graduate School of Medical and Dental Sciences, Tokyo Medical and Dental University, 1-5-45, Yushima, Bunkyo-ku, Tokyo 113-8549, Japan; kasope@tmd.ac.jp (M.K.); arsadr@uw.edu (A.S.); tagami.ope@tmd.ac.jp (J.T.); 2 Department of Operative Dentistry, Graduate School of Medicine, Dentistry and Pharmaceutical Sciences, Okayama University, 2-5-1 Shikata-cho, Kita-ku, Okayama 700-8525, Japan; yoshiyam@md.okayama-u.ac.jp; 3 Biomimetics Biomaterials Biophotonics Biomechanics & Technology Laboratory, Department of Restorative Dentistry, University of Washington, 1959 NE Pacific Street, Seattle, WA 98195-7456, USA; 4 Center of Advanced Medicine for Dental and Oral Diseases, Department for Advanced Dental Research, National Center for Geriatrics and Gerontology, Aichi 474-8511, Japan; yasusumi@ncgg.go.jp

**Keywords:** occlusal tooth wear, erosive tooth wear, SS-OCT, 3D imaging, enamel thickness, dentin exposure, demineralization

## Abstract

The aim of this study was to assess the utility of 3D imaging of optical coherence tomography (OCT) for the diagnosis of occlusal tooth wear ex vivo. Sixty-three extracted human molars with or without visible tooth wear were collected to take digital intraoral radiography and 3D OCT images. The degree of tooth wear was evaluated by 12 examiners and scored using 4-rank scale: 1—slight enamel wear; 2—distinct enamel wear; 3—tooth wear with slight dentin exposure; 4—tooth wear with distinct involvement of dentin. The degree of tooth wear was validated by the histological view of confocal laser scanning microscopy (CLSM). The sensitivity, specificity, and area under the curve (AUC) of receiver operating characteristic analysis were calculated. Diagnostic accuracy was compared with the agreement with CLSM observation using weighted kappa. The results were statistically analyzed at a significance level of α = 0.05. Three-dimensional OCT showed significantly higher sensitivity (*p* < 0.05) for all the diagnostic thresholds of enamel wear and dentin exposure than digital radiography (0.82, 0.85, and 0.79 vs. 0.56, 0.52, and 0.57, respectively). Three-dimensional OCT showed higher AUC and kappa coefficients than digital radiography (*p* < 0.05), where mean AUC and Kappa values were 0.95 and 0.76 for OCT and 0.92 and 0.47 for radiography, respectively. No significant difference of specificity was observed (*p* > 0.05). Three-dimensional OCT could visualize and estimate the degree of tooth wear and detect the dentin exposure at the tooth wear surface accurately and reproducibly. Consequently, a new guideline for tooth wear assessment can be proposed using OCT.

## 1. Introduction

Tooth wear is defined as the loss of dental hard tissue by physical or chemical factors that occur throughout life [1]. The progression of tooth wear is irreversible and its prevalence increases with age [2,3]. The literature considers tooth wear resulting from a multifactorial etiology with interactions of physical and chemical agents. Although the changes resulting from physiological tooth wear are usually subtle and asymptomatic, excessive physical or chemical stimulation can lead to abnormal tooth loss at a pathological level, which may require treatment [3,4]. As healthy life expectancy is essential in an aging society, the importance of long-term maintenance of healthy and functional teeth is increasingly recognized. Advanced tooth wear leads to oral symptoms such as pain and loss of masticatory function and esthetics.

Tooth wear mainly manifests as erosion, attrition, abrasion, or a combination of these factors [1,5]. Erosion is defined as pure chemical wear in the absence of bacteria. Attrition is defined as mechanical wear resulting from tooth–tooth contact like bruxism, whereas abrasion is the loss of tooth substance by repeated physical means other than opposing teeth [6,7]. Erosion results in the softening of enamel and dentin; erosion, together with mechanical wear, facilitates tooth loss to cause pathological wear. Erosion-facilitated tooth wear, defined as “erosive tooth wear” or ETW, may compromise the health of individual teeth and of the entire dentition [8,9]. The prevalence of erosive tooth wear varies widely among populations; however, this condition increasing in younger adults has raised concern in the dental community [10].

In ETW, the impact of dietary or gastric acids in conjunction with attrition or abrasion can affect the occlusal plane of more than one tooth and lead to changes in the shape and size of teeth [9]. Moreover, the dentin surface in ETW involves the exposure of dentin tubules, which can highly initiate dentin hypersensitivity (DH) [11]. For ETW, the speed of tissue loss is mostly at a pathological level, resulting in the inability of the pulp to protect against the stimuli, highlighting the importance of early diagnosis and intervention, including identifying risk factors and discussing with patients to assess and reduce the risk. However, the early signs of enamel wear are difficult to identify in clinic due to the lack of an objective method to diagnose the degree of tooth wear [12]. Visual examination, along with dental radiography, have been frequently employed for the diagnosis of tooth wear and various ETW indices have been introduced. However, a more effective methodology appears necessary to characterize tooth wear from both diagnostic and management aspects.

Optical coherence tomography (OCT) is a noninvasive imaging method that uses interferometry to create a cross-sectional image of optical scattering from internal microstructures [13,14]. Previous studies showed that the recent OCT system has a high degree of sensitivity for detection of dental caries [15,16,17], tooth cracks [18], and interfacial microgaps in adhesive restorations [19,20,21,22]. OCT can also offer remarkable advantages for the measurement of remaining enamel thickness at the tooth wear surface [23,24,25,26]. However, diagnosis of dentin involvement in tooth surface wear is highly required for the prevention and management of the diseases, especially for pathological tooth wear with dentin hypersensitivity. Swept-source OCT (SS-OCT) is a variant of OCT, in which the light source is a tunable laser that sweeps near-infrared wavelength light within millisecond scan delays at kilohertz rates to achieve real-time imaging. High-speed OCT imaging provides an increased number of cross-sectional images that are acquired in a sequence for the generation of three-dimensional (3D) volumetric datasets. Currently, 3D imaging technology for OCT is being utilized in the field of dentistry and has shown excellent results with regard to the analysis of caries, tooth cracks, and marginal defects under resin composite restorations [27].

The aims of this study were to evaluate the diagnostic accuracy of 3D imaging of SS-OCT for tooth wear and to compare the results with those of digital dental radiography. The null hypotheses of this study tested were that 3D OCT could not provide an image for diagnosis of tooth wear level and would have no difference in diagnostic performance of tooth wear compared with conventional methods using visual inspection and radiography.

## 2. Materials and Methods

### 2.1. Specimen Collection

Ethical approval for this study design was obtained from the Institutional Review Board of Tokyo Medical and Dental University (approval number D2013-022). Extracted erupted human molars stored at 4 °C in water containing 0.02% sodium azide were used in this study. Sixty-three molars with or without evidence of tooth wear at occlusal surfaces were collected. The teeth that had visible caries, restoration, or distinct cracks on the occlusal surface were excluded for this study.

The selected teeth were cleaned using a hand scalar and brush cone attached to a low-speed handpiece with a prophylaxis paste. After the removal of calculus or debris, the area of interest for the evaluation of tooth wear level was selected from the occlusal surface for each tooth. Digital photographs of occlusal surface, including the area of interest, were obtained with a digital camera (Nikon D50, Nikon, Tokyo, Japan) with an AF-S Micro Nikkor 105 mm lens with a fixed magnification of ×1.

### 2.2. Digital Dental Radiography

Digital dental radiographs of each tooth were captured with the X-ray tube positioned toward the buccal surface and horizontal aspect of the occlusal surface. A digital intraoral sensor was placed on the lingual aspect of the tooth, and an X-ray unit (Dentnavi Hands, Yoshida Dental, Tokyo, Japan) was operated at exposure settings of 60 kV and 7 mA, with an average exposure time of 0.63 s. Images were viewed on the monitor using the associated image program software (ClinicalView ver. 10.1, Instrumentarium Kavo Dental).

### 2.3. SS-OCT System

After taking digital dental radiographs, SS-OCT scanning was projected onto the occlusal surface including the area of interest to construct 3D images. The prototype SS-OCT system (Yoshida Dental, Tokyo, Japan) used in this experiment employed a frequency domain technique that measured the magnitude and time delay of reflected light to construct a depth profile. The wavelength ranged from 1240 to 1380 nm, with a central wavelength of 1310 nm and a sweep rate of 50 kHz. The high-speed frequency-swept laser was projected onto the occlusal surface using a hand-held scanning probe at a fixed distance to take 3D images. The power of the object beam was 18 mW and the system had a sensitivity of 100 dB. The optical resolution of the 3D dataset in air was less than 11 μm in depth and 40 μm in lateral and axial dimensions.

### 2.4. Scoring by Examiners

Twelve dentists were recruited (8, 9, 10, 11, and 12, with less than 5 years of clinical experience; 6 and 7, with 5–10 years of clinical experience; 3, 4, and 5, with 11–20 years of clinical experience; 1 and 2, with over 21 years of clinical experience) and participated in this experiment as examiners. In order to reach a consensus on the evaluation criteria, the reference examiner (MK) discussed the radiographs and OCT images with 12 dentists in a 1 h session. For the calibration session, MK used 12 extracted teeth images that were not included in the study.

After the discussion, the examiners performed scoring of the tooth wear level of the occlusal surface independently using the following 4-rank scale:

Score 1: Slight enamel wear. Initial tooth wear was within the enamel and more than 1/2 thickness of enamel left.
Score 2: Distinct enamel wear. Tooth wear was within the enamel and less than 1/2 thickness of enamel left.
Score 3: Tooth wear with slight dentin exposure. Tooth wear reached to the dentin-enamel junction (DEJ). Dentin exposure was slight with less than 1 mm diameter.
Score 4: Tooth wear with involvement of dentin. Tooth wear was beyond the DEJ to cause dentin exposure. The diameter of dentin exposure was more than 1 mm.

A liquid crystal display monitor was used to display either digital radiographs or 3D OCT images, associated with the occlusal view of digital photographs. For the radiographs, the original image without enhancement of contrast or brightness was used. For OCT, 3D images of occlusal surface as well as the sequence of two-dimensional (2D) tomographic images extracted from the 3D dataset were dynamically displayed in video format using a custom-developed software (KakumaViewer, Yoshida Dental). Display settings such as brightness and contrast were unchanged from the default for all images and examiners.

### 2.5. Confocal Laser Scanning Microscope Observation

In order to validate the level of occlusal tooth wear, histological sectioning was done to observe the occlusal tooth surface directly under confocal laser scanning microscopy (CLSM) (VK-X150 series, Keyence, Osaka, Japan). The desired cross-sectional slides at the area of interest were marked on the teeth and were sectioned and trimmed from the buccal surface along the mesiodistal plane parallel to the tooth axis using a rotary diamond instrument attached to a highspeed handpiece under copious water coolant. The sectioned teeth were further trimmed using 2000-grit silicon carbide paper, followed by polishing with diamond paste down to 1 µm. The cross-sectioned polished surface was ultrasonicated with distilled water for 1 min to remove the polishing debris and was examined with CLSM at 100× magnification.

### 2.6. Statistical Analysis

The statistical analysis was performed using SPSS for Windows version 23 software (IBM, Armonk, NY, USA). In this study, slight enamel wear for score 1 was considered as physiological tooth wear (intact), whereas tooth wear for scores 2, 3 and 4 was considered as at the pathological level. The sensitivity and specificity of 3D OCT and digital radiography for the diagnosis of distinct enamel wear and involvement of dentin exposure were calculated for each examiner by cross tabulation. Sensitivity and specificity can be put into equations as below:

Sensitivity = Number of positives (true positive) in positive test outcomes/Number of positive conditions

Specificity = Number of negatives (true negative) in negative test outcomes/Number of negative conditions

In this study, sensitivity represents the probability of a diagnostic method identifying the tooth wear at a pathological level which in fact has pathological tooth wear. The higher the value of sensitivity, the more correctly the method can detect the pathological tooth wear.

Specificity represents the probability of a diagnostic method identifying the tooth wear at a physiological level which in fact has physiological tooth wear. The higher the value of specificity, the more correctly the method can detect the physiological tooth wear.

Area under the curve (AUC) of receiver operating characteristic (ROC) analysis was also measured using the results of sensitivity and specificity. The ROC curve was created by plotting the true positive (sensitivity) against the false positive rate (1—specificity). Consequently, the ROC curve depicts the relative trade-offs between true positive and false positive. The best possible diagnostic method is 100% sensitivity and 100% specificity with perfect classification yielding a point in the upper left corner. High AUC value represents good classification results of the method.

The diagnostic accuracy for both imaging methods was calculated using the agreement with histological CLSM observation by weighted Kappa. The results were statistically analyzed using the Wilcoxon rank sum test at a significance level of α = 0.05.

## 3. Results

In this study, a total of 63 molars with enamel wear at the occlusal surface were used. We validated the occlusal surfaces by CLSM observation of histological sections at magnification of ×100. As a result, the 63 teeth included 16 teeth with slight enamel wear (score 1), 18 teeth with distinct enamel wear (score 2), 13 teeth with tooth wear with slight dentin exposure (score 3), and 16 teeth with tooth wear with distinct involvement of dentin exposure (score 4).

**Figure 1:**
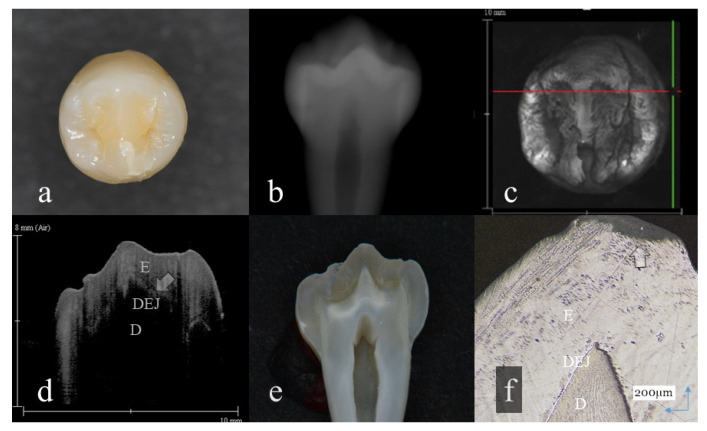
Slight enamel wear (score 1): (**a**): Occlusal view. Visibly, evidence of occlusal tooth wear was not observed; (**b**): Digital intraoral radiography; (**c**): Optical coherence tomography (OCT) en face intensity projection; (**d**): Swept-source (SS)-OCT image. Occlusal enamel and underlining dentin were distinguished by the dentin enamel junction (DEJ) (arrow); (**e**): Histological view; (**f**): Confocal laser scanning microscopy (CLSM) image of histological view. A tip of the cusp was slightly cracked (arrow). However, the anatomical shape of the cusp was maintained. The corresponding dynamic slicing 3D video is in Supplementary Materials: Video S1. The upper right is a cross-sectional image. Lower right is an en face image.

**Figure 2:**
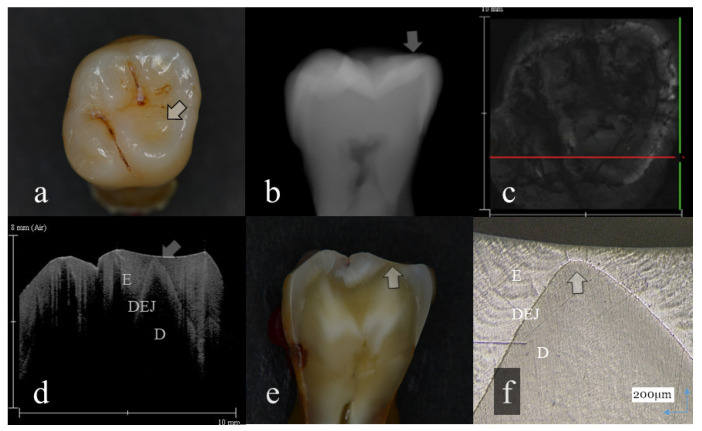
Distinct enamel wear without dentin exposure (score 2): (**a**): Occlusal view. The surface of mesial lingual cusp appeared slightly collapsed (arrow); (**b**): Digital intraoral radiography. Occlusal surface of mesial cusp appeared flattened (arrow); (**c**): OCT en face intensity projection; (**d**): SS-OCT image. Occlusal enamel remaining on the lingual cusp was extremely thin because of the tooth wear. However, dentin was not involved at the tooth wear (arrow); (**e**): Histological view. Remaining enamel at the lingual cusp was extremely thin (arrow); (**f**): CLSM image of histological view. Remaining enamel was observed on the lingual cusp dentin (arrow). The corresponding dynamic slicing 3D video is in Supplementary Materials: Video S2. The image on the upper right is a cross sectional view. Lower right is an en face view.

**Figure 3:**
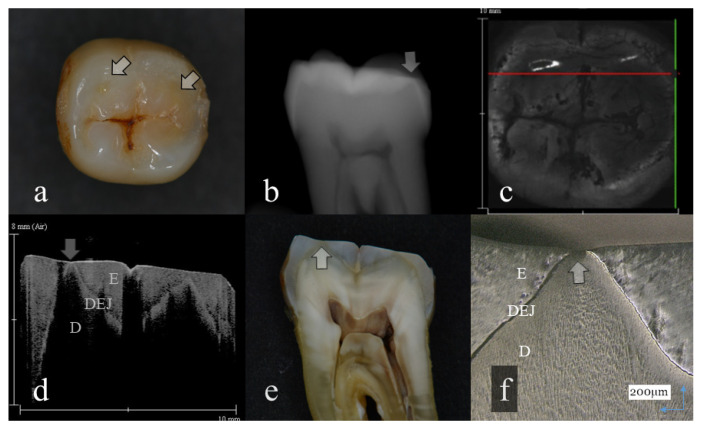
Tooth wear with slight dentin exposure (score 3): (**a**): Occlusal view. The buccal cusps and distal surface were flattened (arrows); (**b**): Digital intraoral radiography. Remaining enamel at the occlusal distal surface was thinned (arrow); (**c**): OCT en face intensity projection; (**d**): SS-OCT image. Occlusal enamel was worn showing the flattened surface. Loss of enamel reached to the DEJ with the slight involvement of dentin (arrow): (**e**): Histological view. Occlusal enamel wear reached to the DEJ depth (arrow); (**f**): CLSM image of histological view. Slight dentin exposure at the cusp was observed (arrow). The corresponding dynamic slicing 3D video in is Supplementary Materials: Video S3. The upper right is a cross sectional image. The image on lower right is an en face image.

**Figure 4:**
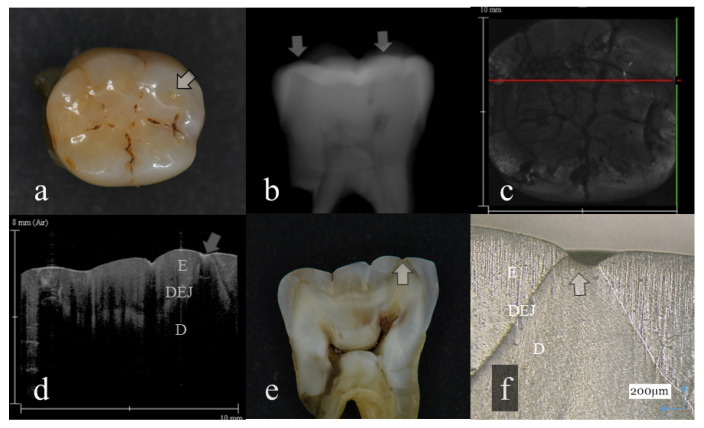
Tooth wear with slight dentin exposure (score 3); (**a**): Occlusal view. Occlusal cusps were flattened. The middle of the cusp was collapsed showing the small dent (arrow); (**b**): Digital intraoral radiography. Remaining enamel thickness of all the tooth crown was extremely reduced (arrow); (**c**): OCT en face intensity projection; (**d**): SS-OCT image. Occlusal enamel was worn, showing the flattened surface. Loss of enamel reached to the DEJ with slight dentin exposure (arrow). The brightness of the exposed dentin surface was increased, suggesting dentin demineralization (arrow); (**e**): Histological view. Enamel thickness for all the tooth crown was thin. Occlusal tooth wear involved slight dentin exposure (arrow); (**f**): CLSM image of histological view. Slight dentin exposure at the cusp was observed (arrow). The corresponding dynamic slicing 3D video is in Supplementary Materials: Video S4. The upper right is a cross sectional view. Lower right is an en face image.

**Figure 5:**
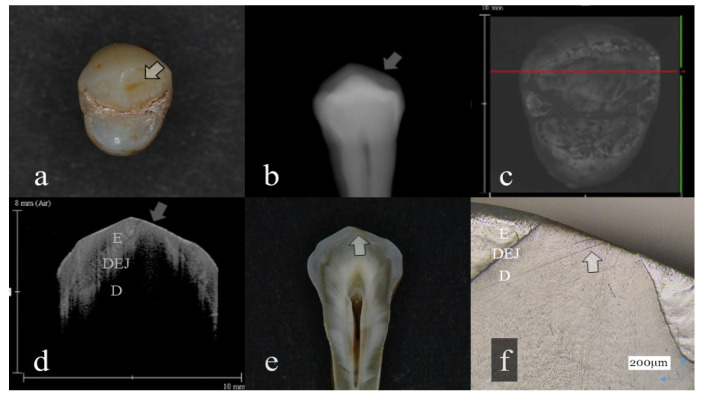
Tooth wear with distinct dentin exposure (score 4); (**a**): Occlusal view. Distal surface of buccal cusp was flattened with a brownish dot (arrow); (**b**): Digital intraoral radiography. In this radiography, buccal and lingual cusps were overlapped to disturb the tooth wear evaluation (arrow); (**c**): OCT en face intensity projection; (**d**): SS-OCT image. The tooth wear with the involvement of dentin was clearly discriminated by the loss of enamel and DEJ continuity (arrow); (**e**): Histological view. Loss of enamel with dentin exposure was evident (arrow); (**f**): CLSM image of histological view. The cusp dentin was exposed to the tooth wear surface (arrow). Evidence of tooth demineralization or enamel cracks was not observed in this case. The corresponding dynamic slicing 3D video is in Supplementary Materials: Video S5. The image in the upper right is a cross sectional view. Lower right is an en face image.

**Figure 6:**
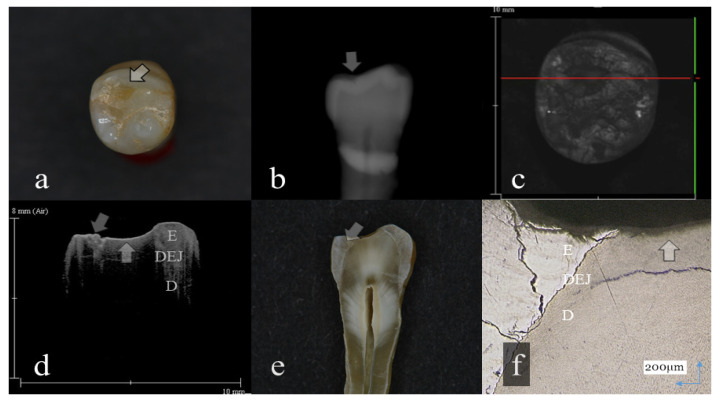
Tooth wear with distinct dentin exposure (score 4); (**a**): Occlusal view. The buccal surface was collapsed to show the scabrous appearance (arrow); (**b**): Digital intraoral radiography. Although the occlusal surface appeared flattened, evaluation of tooth wear level was difficult because of the overlapped image (arrow): (**c**): OCT en face intensity projection; (**d**): SS-OCT image. The tooth wear for both enamel and dentin was clearly imaged. Brightness of exposed dentin subsurface was increased, suggesting dentin demineralization (arrow). The distinct white zone penetrating along DEJ was an enamel crack (arrow); (**e**): Histological view. Tooth wear for both enamel and dentin was evident. The subsurface enamel near DEJ was cracked (arrow); (**f**): CLSM image of histological view. Many crack lines in the enamel were penetrated with changing the direction to reach the DEJ. Subsurface dentin appeared demineralized (arrow). The corresponding dynamic slicing 3D video is in Supplementary Materials: Video S6. The upper right is a cross sectional image. Lower right is an en face image.

**Figure 7:**
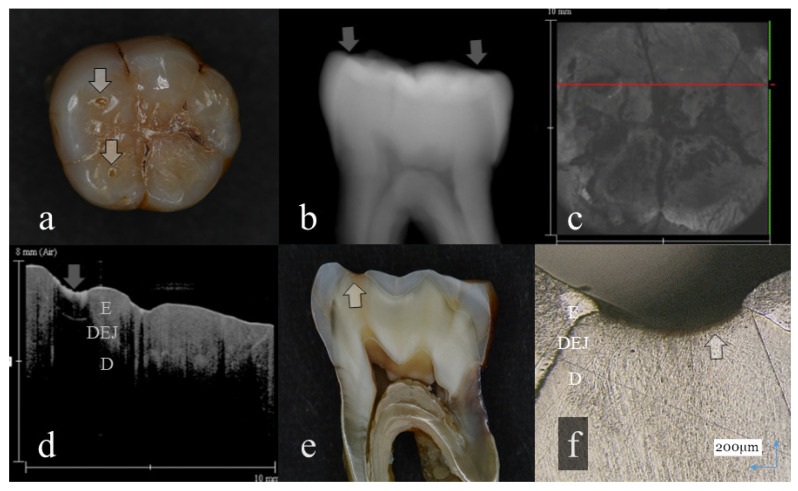
Tooth wear with distinct dentin exposure (score 4); (**a**): Occlusal view. Occlusal cusps were flattened and small dents were observed at the mesial cusps for both the buccal and lingual surface (arrow); (**b**): Digital intraoral radiography. Occlusal surface appeared flattened with the reduced remaining enamel thickness (arrow); (**c**): OCT en face intensity projection; (**d**): SS-OCT image. The tooth wear with distinct dentin exposure was clearly imaged (arrow). Brightness of exposed dentin subsurface was dramatically increased, suggesting the presence of dentin demineralization (arrow); (**e**): Histological view. At the dentin exposure site, the subsurface turned brown (arrow). Remaining enamel thickness of occlusal surface was reduced; (**f**): CLSM image of histological view. Dentin exposure with dentin demineralization was observed at the tooth wear surface (arrow). The corresponding dynamic slicing 3D video is in Supplementary Materials: Video S7. The upper right is a cross sectional view. The image on the lower right is an en face view.

Representative images of 3D OCT and digital radiography are shown in Figure 1, Figure 2, Figure 3, Figure 4, Figure 5, Figure 6 and Figure 7. The sensitivity, specificity, and AUC values for the diagnosis of distinct tooth wear and involvement of dentin in each diagnostic threshold are shown in Table 1 and Table 2. Three-dimensional OCT showed significantly higher sensitivity than the digital radiography for all the diagnostic thresholds of tooth wear level. The sensitivity of 3D OCT for distinct enamel wear, tooth wear with slight dentin exposure, and tooth wear involving dentin was 0.82, 0.85, and 0.79, respectively, whereas for digital dental radiography, it was 0.56, 0.52, and 0.57, respectively. The specificity of 3D OCT and digital radiography was 0.84 and 0.77, respectively, and no significant difference was observed between the values (*p* > 0.05). Diagnostic accuracy as the agreement with the CLSM histological view showed significantly higher results for 3D OCT than digital radiography (Table 3, *p* < 0.05).

## 4. Discussion

Management of dental conditions is shifting from surgical treatment toward prevention and minimally invasive approaches [28]. Tooth wear is an irreversible process, and tooth loss is no exception for prevention and management. Recently, the prevalence of pathological tooth wear has increased in both the young generation and senior citizens, and patients are unaware of changes until the onset of symptoms such as DH or functional impairment. Accurate methodology for the monitoring of tooth loss is crucial for the prevention and management of tooth wear. In this study, 3D imaging of OCT was performed to evaluate the degree of tooth wear at the occlusal surface of extracted human molars. The null hypothesis of this study was rejected; 3D imaging of OCT for evaluation of tooth wear level improved the diagnosis compared with conventional methods using intraoral radiography in combination with visual inspection.

The 3D images generated by OCT allowed for detailed examination of occlusal tooth wear by using multiplanar visualization of dynamic slicing (Figure 1, Figure 2, Figure 3, Figure 4, Figure 5, Figure 6 and Figure 7, Videos S1–S7). The mathematical algorithm of 3D imaging installed in the OCT device appears to improve visualization of the remaining enamel and DEJ. A raw 3D image is generated as a 3D array of voxels or pixels with a grayscale range from 0 to 65,535 in a 16-bit pixel case. Three-dimensional imaging requires defined object boundaries, especially for the creation of 3D surface models by signal filtering. In the OCT system used in the current study, the noise is smoothed by using a Gaussian function on the image pixels, and the edge intensity and edge direction are extracted by an edge extracting filter. The image segmentation of pixel detection mathematically appears to minimize artifacts such as speckle noise, whereas images obtained by coherent imaging systems are inherently corrupted.

In this study, we classified the level of tooth wear into four categories; (1) slight enamel wear, (2) distinct enamel wear, (3) tooth wear with dentin exposure, and (4) tooth wear involving dentin. As tooth wear involving physiological conditions progresses naturally with aging, we presumed the slight enamel wear (score 1) as the physiological tooth wear level. Tooth wear having a score more than level 2 was presumed as at the pathological level and was evaluated by 3D OCT and digital dental radiography. Our results showed that 3D OCT could discriminate the distinct enamel wear and detect the involvement of dentin with significantly higher values of sensitivity, specificity, AUC, and agreement with histology than the radiography (Table 1, Table 2 and Table 3).

In OCT, amount of enamel loss could be estimated by the cross-sectional view of remaining enamel thickness, as the DEJ was clearly visualized as a landmark in all the OCT scans (Figure 1 and Figure 2). Since intact enamel allows the OCT signal to penetrate deep with less scattering, the whole thickness of occlusal enamel could be monitored in this study, resulting in the higher agreement with histological findings (Table 3). These results correlate well with previous study findings measuring the remaining enamel thickness of tooth wear surfaces using cross-polarization (CP) OCT [25,26]. The CP-OCT measurement of remaining enamel thickness was reported to show excellent agreement with the μ-CT measurement [25,26]. Meanwhile, as the enamel thickness is highly location dependent, 3D OCT could visualize the remaining enamel thickness for the area of scanned tooth surface to find the position with the remaining enamel becoming thinner, without the need for additional scanning (Videos S1 and S2). Consequently, dentists allow finding of the location where therapeutic management is necessary, even for patients at the asymptomatic stage. In radiography, level of enamel wear is difficult to see because of the superimposition of dense buccal and lingual cusps, and visual inspection from the occlusal view estimating the topographical and color changes appears more informative in many cases.

Three-dimensional OCT could detect the presence of dentin exposure within the tooth wear surface with pinpoint accuracy (Figure 3, Figure 4, Figure 5, Figure 6 and Figure 7, Videos S3–S7). Since dentin exposure may cause DH in response to mechanical and chemical stimuli during masticatory function, accurate diagnosis for dentin involvement appears beneficent for further maintenance and an intervention approach. Dentin contains 50 vol% of organic structure and scatters light, and signal attenuation of the OCT image through the dentin is higher than the enamel [29]. Optical dissimilarity between the two structures facilitates the discrimination of the tooth wear with and without dentin involvement. In this study, 3D OCT could clearly image the dentin exposure within the tooth wear surface, resulting in high diagnostic capacity and accuracy (Table 1, Table 2 and Table 3). OCT could also facilitate the characterization of exposed dentin surface changes, including the formation and thickness of transparent dentin [29].

In OCT, some of the samples with distinct tooth wear showed increased brightness at the superficial enamel or dentin, suggesting the presence of surface demineralization (Figure 4, Figure 6 and Figure 7) [16,17]. The incidence of demineralization was associated with tooth wear involving dentin (level 4) in many cases. Meanwhile, as the history of extracted teeth was unknown in this study, increasing the frequency of tooth demineralization with the advancement of tooth wear level may suggest the risk of erosion and ETW to facilitate tooth loss to the pathological level. Moreover, it is noteworthy that OCT could detect the enamel crack associated with tooth wear (Figure 6). This finding also showed increasing frequency with the advancement of tooth wear level. In this study, 11 cases out of 29 samples of level 4 dentin exposure showed distinct enamel crack penetrating into DEJ, and 2 cases of 29 samples showed slight enamel crack limited within the enamel thickness.

It is widely recognized that DEJ plays a key role as a damper to transfer loads from the enamel to dentin with different mechanical characteristics. Enamel cracks do not propagate into dentin due to the DEJ, which suppresses crack growth [30,31,32]. On the other hand, enamel tufts are hypocalcified wavy tissue near DEJ and are believed to be the source of cracks during heavy or continuous loading [33,34]. Several lines of evidence showed that dentin is more susceptible than enamel to erosion and abrasion alone or combined [35]. Although the critical pH at which oral fluid is saturated with calcium and phosphate from the tooth surface is not a constant, that for enamel is generally 5.5, yet the critical pH for dentin is 6.5 [36,37,38]. Therefore, once the dentin is exposed, the surface is vulnerable to erosion and can be eroded faster than the enamel. Moreover, the demineralization process can take place along the DEJ to cause an interfacial gap between the enamel and dentin (Figure 6). Since tooth wear involving dentin reveals DEJ to the oral environment, the superficial enamel adjacent to the dentin exposure site may lose the underlying support of dentin due to the erosion along DEJ. The presence of superficial demineralization and enamel cracks at the tooth wear surface may accelerate the lesion progress and suggest the difficulty of clinical management and intervention for pathological tooth wear. These results also imply that subsurface demineralization and cracks should be taken into consideration for the prognosis of restorations for tooth wear surfaces. Consequently, accurate diagnosis of occlusal tooth wear regarding the depth and subsurface condition appears crucial for the determination of therapeutic approach. From this viewpoint, OCT appears to be a suitable method to monitor and manage the tooth wear.

Based on the results of this study, 3D OCT could provide images for diagnosing the level of tooth wear to improve the diagnostic accuracy of tooth wear compared with traditional methods using visual inspection and radiography. On the other hand, the rate of enamel loss in low pH was reported to speed up and occur faster than dentin, especially under the condition simulating mechanical action and gastric reflux [39,40]. Consequently, accurate diagnosis of early signs for tooth wear and ETW is essential to prevent further loss of tooth structure and pathological tooth wear. Within the limitation of this ex vivo study, 3D OCT can potentially help in providing valuable information for the remaining enamel thickness, amount of dentin exposure as well as enamel crack. Further study is necessary for clinical applications of OCT for tooth wear detection and monitoring, and for the management of pathological tooth wear.

## 5. Conclusions

3D OCT could image the degree of enamel loss and dentin exposure for occlusal tooth wear without the risk of X-ray exposure. The presence of tooth demineralization and enamel cracks involved in the tooth wear surfaces were also imaged in OCT. Three-dimensional OCT appears to be a safer option for the monitoring and management of tooth wear to prevent further tooth loss at the pathological level. Future clinical studies are required to image pathological tooth wear in vivo.

## Figures and Tables

**Table 1:** Sensitivity (score 2, score 3, and score 4), specificity, and AUC values, obtained from SS-OCT.

Investigator	Sensitivity			Specificity	AUC
	Score 2	Score 3	Score 4		
**1**	0.83	1.00	0.56	0.60	0.93
**2**	0.78	0.85	1.00	0.94	0.98
**3**	0.83	0.92	0.88	0.81	0.97
**4**	0.94	0.69	0.63	0.88	0.94
**5**	0.78	0.38	0.63	0.63	0.82
**6**	0.83	1.00	0.75	0.81	0.96
**7**	0.72	1.00	0.63	0.81	0.94
**8**	0.83	0.85	0.88	0.94	0.96
**9**	0.72	0.92	0.69	1.00	0.95
**10**	0.83	0.69	1.00	0.81	0.96
**11**	0.83	0.85	0.88	0.88	0.98
**12**	0.89	1.00	0.94	1.00	0.99
Mean	0.82	0.85	0.79	0.84	0.95
S.D.	0.06	0.18	0.16	0.13	0.04

**Table 2:** Sensitivity (score 2, score 3, and score 4), specificity, and AUC values, obtained from digital dental radiograph.

Investigator	Sensitivity			Specificity	AUC
	Score 2	Score 3	Score 4		
**1**	0.78	0.77	0.38	0.50	0.92
**2**	0.50	0.31	0.63	0.81	0.93
**3**	0.50	0.62	0.69	0.69	0.93
**4**	0.61	0.46	0.50	0.81	0.91
**5**	0.56	0.31	0.63	0.81	0.93
**6**	0.72	0.69	0.50	0.63	0.92
**7**	0.22	0.46	0.31	0.75	0.92
**8**	0.56	0.62	0.56	1.00	0.92
**9**	0.33	0.62	0.69	0.81	0.93
**10**	0.72	0.38	0.81	0.94	0.93
**11**	0.61	0.46	0.56	0.75	0.91
**12**	0.56	0.54	0.56	0.69	0.90
Mean	0.56	0.52	0.57	0.77	0.92
S.D.	0.16	0.15	0.14	0.13	0.01

**Table 3:** Diagnostic agreement with CLSM (weighted Kappa).

	Mean (S.D.)
SS-OCT	0.76(0.12)
Digital dental radiograph	0.47(0.09)

## Supplementary Materials

The following are available online at https://www.mdpi.com/1424-8220/20/21/6016/s1, Video S1: 3D image of Figure 1 with dynamic slicing (Score 1); Video S2: 3D image of Figure 2 with dynamic slicing (Score 2); Video S3: 3D image of Figure 3 with dynamic slicing (Score 3); Video S4: 3D image of Figure 4 with dynamic slicing (Score 3); Video S5: 3D image of Figure 5 with dynamic slicing (Score 4); Video S6: 3D image of Figure 6 with dynamic slicing (Score 4); Video S7: 3D image of Figure 7 with dynamic slicing (Score 4).
